# An Index of International Dental Literature

**Published:** 1912-07-15

**Authors:** 


### AN INDEX OF INTERNATIONAL DENTAL LITERATURE.

The need for a dental bibliography and guide to periodical literature was a much discussed theme at the International Odontological Congress in Berlin in 1909. Stress was laid upon the very rapid progress made by dental science during the last decade, the fact that that progress may be materially facilitated by international co-operation both in matters of theory and in the results of research and of practice. The importance of the function of literature in dentistry can hardly be over-estimated. But, while each nation has its own text-books and journals, it is still difficult for the individual, even though he have the gift of tongues, to find out what is going on in other countries.

Since the publication in New York from 1839-1849 of the *American Library of Dental Science* there have been many similar works, the best being Ports' *Index of German Dental Literature and German Bibliography*. But that came to an end in 1902.

What is needed is an International Index published periodically, and this at last has been begun by Dr. Paul de Terra, under the *aegis* of Dr. Guido Fischer, and is now to be finally established in the columns of the international numbers of the *Deutsche Zahnarztliche Zeitung* under the title of *Index Stomatologicus*. It will appear monthly and will be compiled from sixty-five journals and periodicals and will include works on all subjects related to dentistry—and a carefully classified list of subject headings. The items are cited in their respective languages as well as in German. Russian is excepted. In the first number there are three hundred and thirty-four items arranged according to twenty-four subject headings.—Abstracted from the *Deutsche Zahnarztliche Zeitung*.

